# InSAR Baseline Estimation for Gaofen-3 Real-Time DEM Generation

**DOI:** 10.3390/s18072152

**Published:** 2018-07-04

**Authors:** Huan Lu, Zhiyong Suo, Zhenfang Li, Jinwei Xie, Jiwei Zhao, Qingjun Zhang

**Affiliations:** 1 National Laboratory of Radar Signal Processing, Xidian University, Xi’an 710071, China; luhuanohyeah@126.com (H.L.); lzf@xidian.edu.cn (Z.L.); jerryxie9@gmail.com (J.X.); 2 Institute of Space-Terrestrial Intelligent Networks (ISTIN) Group, Nanjing University, Nanjing 210023, China; jackokie@gmail.com; 3 Beijing Institute of Spacecraft System Engineering, China Academy of Space Technology, Beijing 100094, China; zhangqj@cast.cn

**Keywords:** GF-3, InSAR, DEM, baseline estimation, real-time orbit

## Abstract

For Interferometry Synthetic Aperture Radar (InSAR), the normal baseline is one of the main factors that affects the accuracy of the ground elevation. For Gaofen-3 (GF-3) InSAR processing, the poor accuracy of the real-time orbit determination results in a large baseline error, leads to a modulation error in azimuth and a slope error in the range for timely Digital Elevation Model (DEM) generation. In order to address this problem, a novel baseline estimation approach based on Shuttle Radar Topography Mission (SRTM) DEM is proposed in this paper. Firstly, the orbit fitting is executed to remove the non-linear error factor, which is different from traditional methods. Secondly, the height errors are obtained in a slant-range plane between SRTM DEM and the GF-3 generated DEM, which can be used to estimate the baseline error with a linear variation. Then, the real-time orbit can be calibrated by the baseline error. Finally, the DEM generation is performed by using the modified baseline and orbit. This approach has the merit of spatial and precise orbital free ability. Based on the results of GF-3 interferometric SAR data for Hebei, the effectiveness of the proposed algorithm is verified and the accuracy of GF-3 real-time DEM products can be improved extensively.

## 1. Introduction

The baseline is an important parameter in Interferometry Synthetic Aperture Radar (InSAR) that affects the accuracy of the surface elevation, which is defined as the distance between the two antenna phase centers that illuminate the same area [1]. The accuracy of calibration parameters plays an important role in real-time Digital Elevation Model (DEM) generation in some special conditions, such as natural disasters. Hence, it is a great challenge to improve the accuracy of the baseline which is taken as an important parameter in calibration.

A variety of estimation algorithms have been studied to improve the accuracy of the baseline. So far, many satellite-based InSAR baseline estimation methods have been studied. The basic and typical algorithms [2] of baseline estimation are the satellite orbit state vector method, the external control points method and the Fast Fourier Transformation (FFT) method.

The satellite orbit state vector method uses the satellite ephemeris parameters to obtain baseline parameters through the vector difference between the two satellite positions. However, the precision of the baseline cannot be guaranteed because it is affected greatly by satellite positioning. Zheng et al. [3] proposed a method of orbital linear fitting to satisfy the precision of space borne InSAR interference measurement, which has higher accuracy than the general method by original orbit. Nevertheless, the accuracy of it cannot be guaranteed under the real-time orbit. 

The external control points method uses the ground control points (GCPs) with known elevation information and interferometric phase and combines the equations of the interferometric system to obtain the baseline parameters with high accuracy [4]. Du et al. [5] studied the estimation of baseline parameters of space-borne InSAR based on GCPs and the method was tested with ENVISAT ASAR data for the Dujiangyan region of Sichuan Province of China. A new estimation algorithm with block adjustment considering the phase offset for multiple InSAR data that covers large areas was proposed to reduce the number of ground control points and the difference between heights derived from different data [6]. The experiments of baseline estimation with block adjustment are explored with multiple pairs of InSAR data obtained by airborne InSAR system were experimentally verified by Institute of Electronics, Chinese Academy of Sciences with less ground control points. In [7], an accurate method for determining the precise interferometric baseline was provided with the aid of the least squares estimation by using ground control points. However, the number of GCPs must be enough and the computation is extensive. The method is only applicable to data with GCPs and the scope of application is limited.

The FFT initial estimation method is based on the frequency information of flat interference fringes to determine calibration parameters. Singh et al. [8] proposed a method that the baseline parameters can be retrieved from a Fourier analysis of the interferometric fringes. Xu et al. [9] proposed a novel high precision space borne InSAR baseline estimation approach based on interferometric fringe frequency, which makes improvements from two aspects: the baseline calculation equation and the fringe frequency estimation precision. However, the selected data area should be flat in this method. Therefore, a large measurement error will exist for some uneven areas or special circumstances such as earthquakes.

In addition, a lot of works about baseline estimation have been studied except traditional methods. Jin et al. [10] proposed an initial baseline estimation of InSAR based on the phases of flat earth. A calibration estimation method [11] which uses co-registration offsets and non-linear least square algorithm was proposed but the vertical baseline is inaccurate. Two ENVISAT SLC (Single Look Complex) images covering Tibet, China were applied to test this method. In terms of the vertical effective baseline, Chen et al. [12] proposed an estimation of InSAR baseline based on the Frequency Shift Theory. In addition, there is an estimation method based on subspace projection [13]. It is highly robust to the unwrapping phase and can restrain the noise well but the information about some points on the ground was used in this method. A new approach for interferometric calibration was proposed [14,15], based on the idea of maximizing the correlation between the original complex interferogram and reference values of it obtained from ground control points. The main advantage with respect to traditional techniques is that the method does not require the phase to be unwrapped in advance and the method is computationally more demanding than traditional techniques.

However, the research angle of these approaches is the physical baseline or vertical effective baseline. Different from them, our method makes use of SRTM DEM to estimate the parallel baseline instead of the physical baseline or vertical baseline. It comes along with the following advantages: the modulation error in azimuth can be demonstrated clearly because its variation tendency is basically in line with the parallel baseline change and the “slope” error in range can be obtained directly. Besides, our method has no need for corner reflectors and no restriction to flat terrain in the scenes. Therefore, the terrain information can be obtained timely and accurately with our method under earthquake conditions.

In this paper, a novel method of baseline estimation based on SRTM DEM for real-time DEM generation is proposed and the effectiveness of the approach is verified using GF-3 satellite data for Hebei area, which is the first time to use this method for GF-3 interferometric processing. First, the preprocessing—which is called orbit fitting—is performed to remove the non-linear error factor according to the characters of GF-3 real-time orbits and there is no pretreatment before estimating calibration parameters in the conventional methods. Then, considering the “slope” effect in GF-3 interferometric processing, the physical baseline is decomposed to analyze the main source of the problem and then the accuracy of the DEM product can be improved by correcting the parallel baseline component instead of the physical baseline. In addition, the error of estimation is linear instead of a constant as opposed to some general methods so that the DEM product is more reliable. Finally, from the scope of application, our method will not be affected by GCPs, topography and orbit accuracy. Therefore, the approach presented herein can obtain terrain information with high accuracy during natural disasters such as earthquakes and it can provide direct data support to help post-earthquake emergency commanders and staff understand the landscape of the earthquake area.

The remainder of this paper is organized as follows: Section 2 explains the InSAR processing chain briefly, then the principle and flow of the baseline estimation for real-time DEM generation by SRTM DEM are given after analyzing the baseline errors. In Section 3, the experiments on two GF-3 SAR images are conducted to validate the effectiveness and efficiency of the proposed method. Finally, the conclusions are drawn in Section 4.

## 2. Principle and Methods

### 2.1. Principle and InSAR Processing

**Figure 1:**
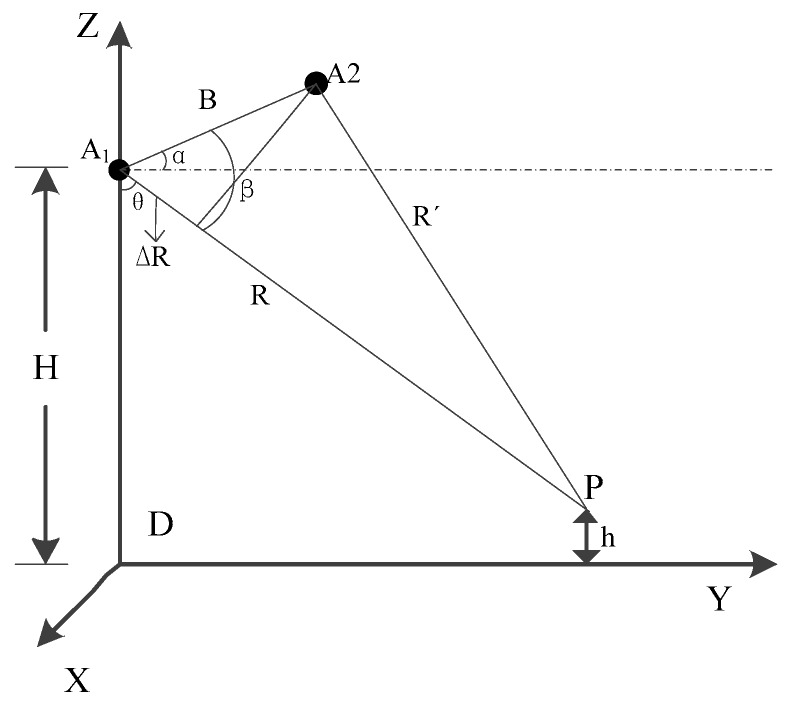
Repeat-pass work mode.

The basic principle of DEM generation [16,17] uses two SAR antennas with interferometric imaging capabilities (or repeatable observation of one antenna) to acquire two coherent single-look complex images in the same region with certain different looking angles. GF-3 satellite is the repeat-pass mode and it is difficult to ensure proper baseline parameters because the ground conditions and scattering characteristics may have changed due to a time interval between two images. The operating mode is shown in Figure 1.

As demonstrated in Figure 1, the elevation of the antenna phase center *A*_1_ is *H* and the elevation of the ground point *P* is *h*. *θ* indicates the side looking angle, *B* denotes the baseline of the distance between the two antennas, *α* is the angle between *B* and the horizontal direction, *R* and *R*′ are the slant-range from the two antenna phase centers to the target point *P* respectively and Δ*R* represents the corresponding slant-range difference.

On the basis of the geometry of Figure 1 and the cosine theorem, the elevation *h* of the ground point *P* is denoted as:

(1) $$h=H-R\cos(90{}^{\circ}+\alpha -\arccos(-\frac{\Delta R}{B}+\frac{B}{2R}-\frac{\Delta R^{2}}{2RB}))$$

**Figure 2:**
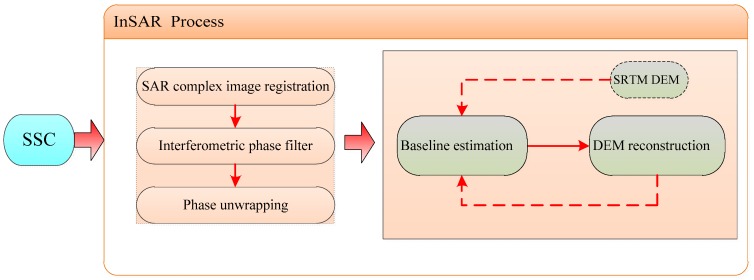
Interferometry Synthetic Aperture Radar (InSAR) technical flow chart.

From the foregoing description, it can be seen that the geometrical principle of InSAR for measuring terrain height is not complicate and it has some problems with the implementation. On the one hand, it is hard to obtain two highly coherent SAR images for the same scene. On the other hand, the true value of the interferometric phase cannot be obtained directly from the complex image, which makes the InSAR process chain become complex. The basic flow for establishing the ground height model by using the interferometric method for elevation measurement is demonstrated in Figure 2.

As shown in Figure 2, after registering the Slant-range Single-look Complex SAR image (SSC), we perform the processing of interferometric phase filter and phase unwrapping on the data transformed [18,19,20,21,22]. Then, the topographic information will be obtained with the accurate baseline. In some special conditions such as earthquake and other natural calamities, DEM generation timely is important. However, the scientific orbital data acquired after tens of days cannot guarantee the timeliness of obtaining the geomorphic information for rescuers. Therefore, the real-time orbit must be corrected in some methods to obtain higher-precision DEM products. It is the key of generating DEM to build a proper model and exactly eliminate the influence of baseline error.

### 2.2. Baseline Error Analysis

**Figure 3:**
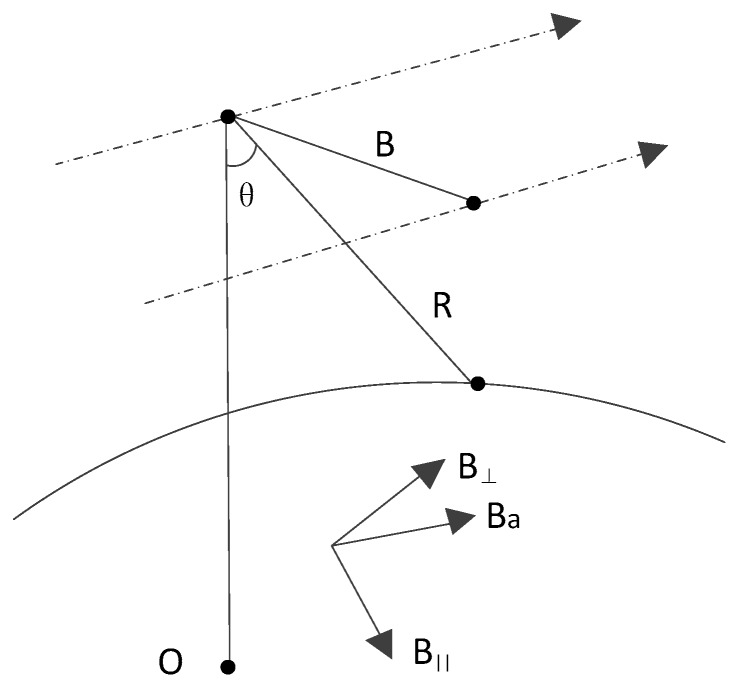
Baseline decomposition manner.

Figure 3 shows the baseline decomposition manner to perform error analysis, among which the three coordinates are along the heading $B_{a}$, along the line of sight direction $B_{∥}^{}$ and the vertical line of sight $B_{⊥}$ respectively.

The image coherence, height sensitivity and the measurement accuracy are affected by baseline error directly [23,24]. The height error introduced by $B_{⊥}$ is in the level of centimeter and can be ignored [25] and the error $\Delta B_{a}$ also can be negligible on height accuracy because the equation which affect the DEM generation are not changed even though there is a measurement error along the heading baseline. However, the baseline error $\Delta B_{∥}$ plays an important role in DEM generation and we will analyze it in detail below.

In space borne interferometric geometry, the baseline length is much smaller than the slant range and $B_{∥}$ equals the one-way wave path-difference of the electromagnetic wave. Therefore, the height error caused by the baseline measurement error of $B_{∥}$ can be denoted as [26]:

(2) $$\Delta h=h_{amb}\frac{2\Delta B_{∥}}{\lambda }$$

From Equation (2), the accuracy of the elevation will be affected by $\Delta B_{∥}$. In addition, the $\Delta B_{∥}$ will bring about a slope error [27] in range because the height of ambiguity $h_{amb}$ varies along the distance. The corresponding slope is defined as:

(3) $$k_{tilt}=\frac{\Delta h_{far}-\Delta h_{near}}{R_{far}\sin\theta_{far}-R_{near}\sin\theta_{near}}=\frac{\Delta B_{∥}}{B_{⊥}}$$

where $R_{near}$ and $R_{far}$ denote the proximal and distal distance respectively, $\theta_{near}$ and $\theta_{far}$ indicate the proximal and distal angles of view, $\Delta h_{far}-\Delta h_{near}$ is the difference between the proximal and distal elevation and $R_{far}\sin\theta_{far}-R_{near}\sin\theta_{near}$ represents the distance between the proximal and distal distance. The elevation error varies in meters and a relative elevation error is generated in a 1°×1° range of DEM (approximate 10^5^ m in ground-range) because of $\frac{\Delta B_{∥}}{B_{∥}}\approx 10^{-5}$.

According to the analysis above and the equations, the main factor which causes DEM precision loss is $\Delta B_{∥}$. Therefore, an appropriate model should be built to correct the $\Delta B_{∥}$ so as to generate a higher precision DEM.

### 2.3. The Proposed Method

**Figure 4:**
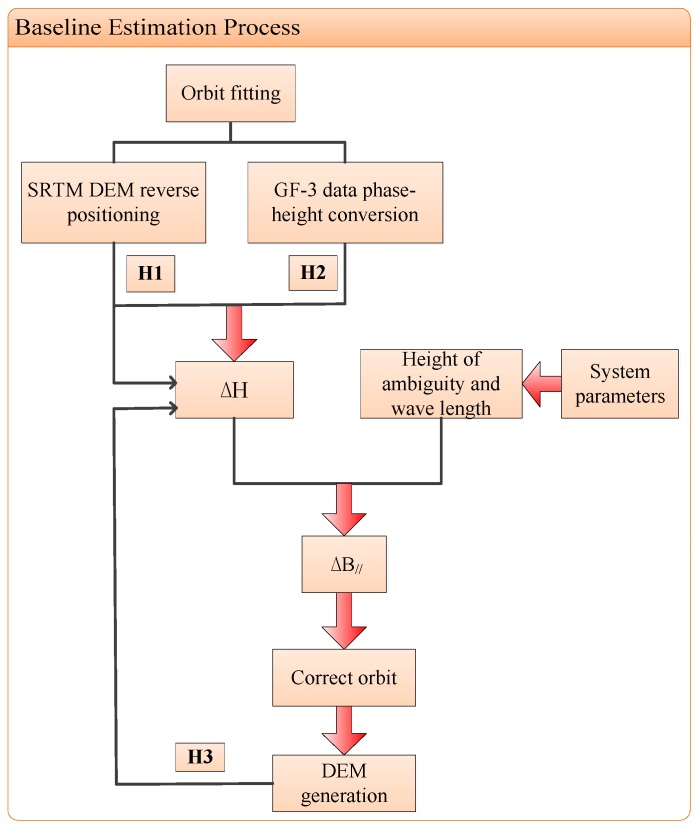
Baseline estimation with Shuttle Radar Topography Mission Digital Elevation Model (SRTM DEM).

Figure 4 displays the process chain of baseline estimation by SRTM DEM. On account of a poor accuracy of the real-time orbit in GF-3 data, orbit fitting is the preprocessing before baseline estimation. In this way, the non-linear error factor will be removed and some rules of the baseline change can be observed clearly. It is critical for obtaining baseline error to acquire the elevation error between SRTM DEM and GF-3 generated DEM in slant-range plane. In addition, in order to acquire a higher precision DEM product, the baseline error correction is critical. After correcting the linear variation error, a more precise orbits and DEM product can be obtained. Finally, the DEM generation is performed which can verify the result of our baseline estimation. The procedure of the proposed approach is given in details.

#### 2.3.1. Orbit Fitting

It is hard to obtain the baseline error accurately due to the low accuracy of the real-time orbit determination of GF-3 data. Therefore, orbit fitting must be performed in order to wipe out the non-linear error factor. The Lagrange polynomial curve fit method [28] with acceptable feasibility and accuracy will be used to perform it. The Lagrange interpolation polynomial can be expressed as follows:

(4) $$\begin{array}{l} L(x)=\sum_{j=0}^{k}y_{j}l_{j}(x)\\ l_{j}(x)=\prod_{i=0,i\neq j}^{k}\frac{x-x_{i}}{x_{j}-x_{i}}=\frac{x-x_{0}}{x_{j}-x_{0}}\cdots \frac{x-x_{j-1}}{x_{j}-x_{j-1}}\frac{x-x_{j+1}}{x_{j}-x_{j+1}}\cdots \end{array}$$

where $x_{j}$ represents the independent variable, $y_{j}$ corresponds to the value of the function at $x_{j}$ and $l_{j}(x)$ is the interpolation cardinal function. After orbit fitting, some inaccurate orbit positions will be calibrated.

#### 2.3.2. Elevation Error in Slant-Range Plane

It shows that the baseline error is closely related to the elevation error from Equation (2). Thus the elevation error between SRTM DEM and the GF-3 generated DEM in slant-range plane must be obtained. The inverse positioning is proposed which includes three steps to obtain the height of SRTM DEM in slant-range plane.

(1) Coordinate conversion

SRTM DEM provides three-dimensional coordinates of the latitude, longitude and elevation in ground-plane, whereas the information for each point in slant-range plane is usually implemented in the WGS84 (World Geodetic System-1984 Coordinate System) coordinate system. Specially, coordinate must be transformed for the sake of finding corresponding positions of SRTM DEM in slant-range plane. The conversion relationship can be displayed as follows:

(5) $$\left\{ \begin{array}{l} X=(N+H)\cos B\cos L\\ Y=(N+H)\cos B\sin L\\ Z=(N(1-e^{2})+H)\sin B\\ N=a{\left(1-e^{2}\sin^{2}B\right)}^{-1/2} \end{array}\right.$$

where *X*, *Y* and *Z* represent the coordinates in WGS84 coordinate system, *B* means north latitude, *L* indicates longitude, *H* is the height, *N* demotes the radius of curvature in prime vertical of the point, *a* illustrates the Semi-Major Axis and *e*^2^ represents the first eccentricity. After the conversion of Equation (5), the matching WGS84 coordinate of each position can be acquired.

(2) SRTM DEM interpolation

Generally speaking, the height matrix size obtained by SRTM DEM is much smaller than the size of GF-3 image. In order to get a more accurate elevation value after the reverse positioning, the elevation matrix generated by the SRTM DEM should be interpolated. In the experiment, the original elevation matrix is interpolated into an elevation matrix of 2 × *Na* × 2 × *Nr* (*Na* and *Nr* are the number of azimuth and range pixels respectively).

(3) Obtain the elevation of SRTM DEM in slant-range plane

The position in azimuth of each point in slant-range plane will be obtained through the focused Doppler center and the location in range will be acquired by combining the sampling interval and slant range from the parameter file. Then the elevation of SRTM DEM in slant-range plane will be received by reverse positioning. Finally, the height matrix should be interpolated based on the azimuth and distance pixels for several inaccurate elevation.

**Figure 5:**
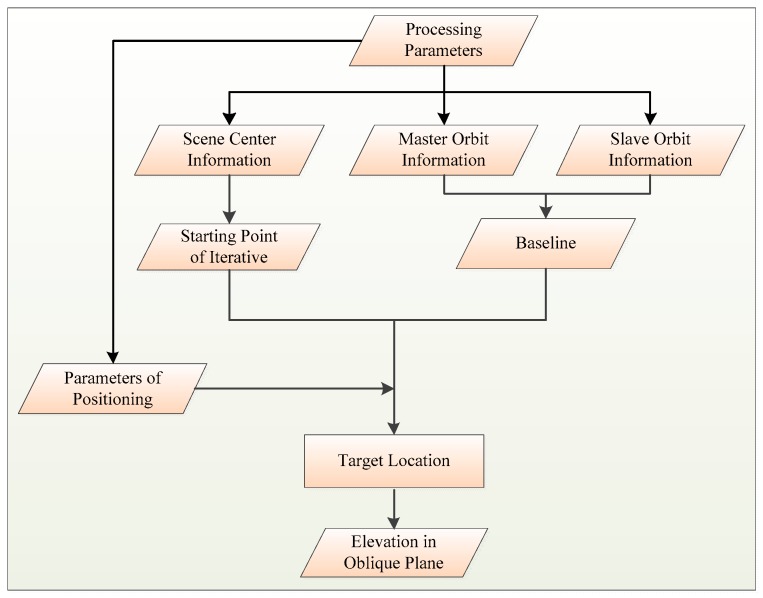
Target location by iterative method.

However, the elevation of GF-3 data in slant-range plane is achieved by means of target location with Newton iterative method [29,30] which is different from the reverse positioning method. The three-dimensional position information of any point in the scene will be obtained by solving the slant range equation, interferometric phase equation and Doppler equation [24,31]. According to InSAR geometric model in Section 2.1, positioning equation can be expressed as follows:

(6) $$\left\{ \begin{array}{c} \left|\vec{S_{1}}-\vec{P}\right|=R_{1}\\ \left|\vec{S_{2}}-\vec{P}\right|=R_{2}\\ f_{dc1}=-2(\vec{S_{1}}-\vec{P})\cdot (\vec{V_{S1}}-\vec{V_{P}})/(\lambda R_{1}) \end{array}\right.$$

where $S_{1}$ is the phase center of the transmitting antenna (namely master antenna), $S_{2}$ is the phase center of the receiving antenna (namely slave antenna), P represents the ground target, λ represents the wavelength, $\vec{V_{S1}}$ indicates the master antenna’s velocity vector, $\vec{V_{P}}$ is the velocity vector of the ground target point and $f_{dc1}$ illustrates the imaging Doppler center frequency of the master antenna. The above equations can be defined as follows:

(7) $$\begin{array}{l} {\left(x_{1}-x\right)}^{2}+{\left(y_{1}-y\right)}^{2}+{\left(z_{1}-z\right)}^{2}=R_{1}{}^{2}\\ {\left(x_{2}-x\right)}^{2}+{\left(y_{2}-y\right)}^{2}+{\left(z_{2}-z\right)}^{2}=R_{2}{}^{2}\\ f_{dc}=-\frac{2\left[(x_{1}-x)V_{x}+(y_{1}-y)V_{y}+(z_{1}-z)V_{z}\right]}{\lambda R_{1}} \end{array}$$

where $\left(x_{1},y_{1},z_{1}\right)$ and $\left(x_{2},y_{2},z_{2}\right)$ are the coordinates of $S_{1}$ and $S_{2}$ respectively, (x,y,z) represents the coordinates of *P* and $\left(V_{x},V_{y},V_{z}\right)$ is the speed of the master antenna. The system of equations is a ternary quadratic system of equations that can be solved using the Newton iteration method. Then we can get the true ground coordinates and elevation of the target point and recover the real terrain through grid interpolation. The implementation flow chart is exhibited in Figure 5.

#### 2.3.3. Baseline Error Estimation

As can be seen in Equation (2), the height of ambiguity is written as:

(8) $$h_{amb}=\frac{\lambda R\sin\theta }{2B_{⊥}}$$

From Equation (2), the $\Delta B_{∥}$ will be acquired by the elevation error along the azimuth, the height of ambiguity and the wave length. The $\Delta B_{∥}$ is fitted by using least-square. Therefore, $\Delta B_{∥}$ shows a linear variation instead of a constant estimated. It is worth noting that the error is not under ECR (Earth-Fixed Coordinate System) coordinate system, while the orbit position is obtained under the ECR coordinate system. Therefore, it is necessary to implement coordinate conversion when correcting the orbit by baseline error.

#### 2.3.4. Orbit Correction

**Figure 6:**
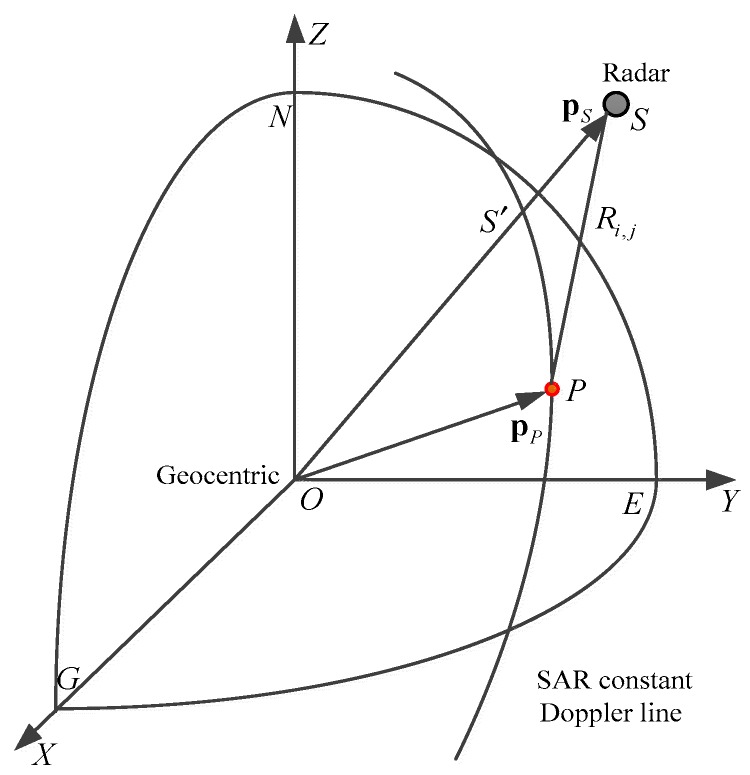
ECR coordinate system.

The coordinate system of orbits is showed in Figure 6. *O*-*XYZ* denotes the ECR coordinate system, *N* represents the North Pole, *Z* axis coincides with the earth’s rotation axis and *S* represents the radar antenna whose position vector and radar velocity vector are $p_{S}=(x_{1},y_{1},z_{1})$ and $v_{s}=(v_{x},v_{y},v_{z})$ respectively. The position of the sub-satellite point is *S*′, and *P* represents the ground target whose position vector is $p_{p}=(x,y,z)$.

On the basis of the baseline vector coordinate system that is demonstrated in Figure 3, we convert $\Delta B_{∥}$ to the ECR coordinate system and obtain the triaxial component. And the orbit will be calibrated based on this component.

In this paper, the accuracy of the DEM product is used as an indicator to verify the baseline correction. Therefore, DEM generation should be performed after baseline and orbit correction.

#### 2.3.5. DEM Generation

DEM is the main product of InSAR processing [32]. DEM generation consists of two parts: target location and geocoding [33,34]. Target location has already been introduced as mentioned earlier. The geocoding process projects the elevation on the slant-range plane into the ground-range plane to provide the final DEM product. In fact, DEM generation is the criteria of baseline estimation. 

In order to represent the difference between our method and the traditional methods more directly, we show four different aspects of processing of the baseline estimation, that is, the baseline form, preprocessing, the baseline error and limiting conditions, to carry out a system and comparative study. In terms of the research of baseline form, in general, the traditional baseline estimation calculates the physical baseline *B* and the horizontal angle *α*. The parallel baseline will be estimated in our method because it is the key source of “slope” effect in GF-3 interference processing. The second difference is that there is no preprocessing in other approaches. Then, what is noteworthy is that the error is a constant in other methods, while it is linear in our approach. In addition, our approach has no limits of orbits, GCPs and terrain. Table 1 shows the details of comparison between the proposed approach and three conventional methods. 

## 3. Experimental Results

### 3.1. Experimental Data

**Figure 7:**
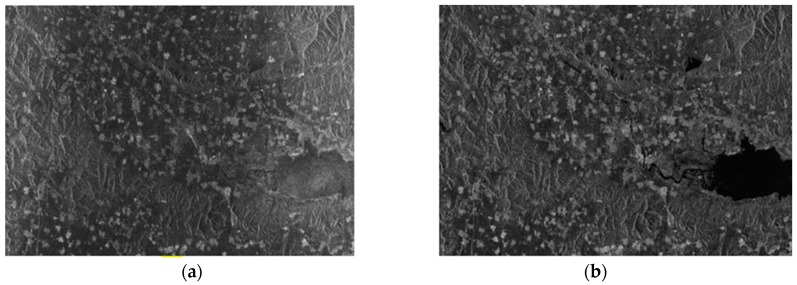
Experimental data: (**a**) the master image of the experiment; (**b**) the slave image of the experiment.

In order to achieve the InSAR process effectively, image pairs in the same area must have good coherence. After extensive selection and testing of GF-3 SAR images, a pair of images are selected after interception of the public area, which are shown in Figure 7.

The main parameters of the data used in the experiment are shown in Table 2.

### 3.2. Results of Baseline Estimation

#### 3.2.1. Results of No Correction

**Figure 8:**
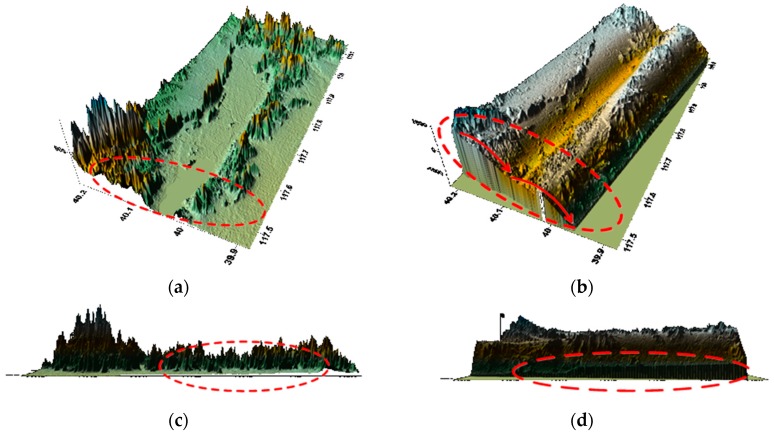
DEM product without any correction: (**a**) SRTM DEM along the latitude; (**b**) GF-3 generated DEM along the latitude; (**c**) SRTM DEM along the longitude; (**d**) GF-3 generated DEM along the longitude.

Figure 8 shows the result of GF-3 DEM generation without any correction and the comparison with SRTM DEM.

As can be seen in Figure 8, both latitude and longitude directions have errors if there is no correction and a large elevation error exists while comparing with SRTM DEM. 

#### 3.2.2. Results of Baseline Error

**Figure 9:**
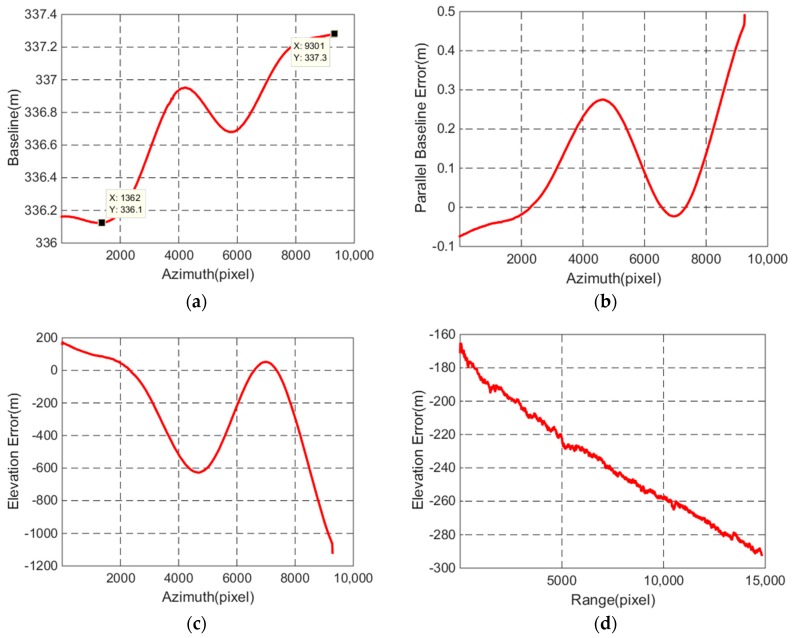
Different error curves: (**a**) description of the baseline length; (**b**) description of the parallel baseline error; (**c**) description of the vertical error in azimuth between SRTM DEM and the GF-3 generated DEM; (**d**) description of the vertical error in range between SRTM DEM and the GF-3 generated DEM.

According to Figure 8a, different error curves which are shown in Figure 9 can be obtained over the entire scene.

As can be seen from the two points marked in Figure 9a, the baseline changes reach to 1.2 m. From Figure 9b, the parallel baseline error is up to 10^−1^ m, which is too large to generate a high-precision DEM. The elevation error curve in azimuth in Figure 9c shows the same tendency as the elevation changes in azimuth in Figure 8b, while it exhibits and an approximately linear change in range in Figure 9d. All the results in Figure 9 indicate that the baseline error will lead to the slope error in range and the modulation error in azimuth. 

#### 3.2.3. Results of Orbit Fitting

**Figure 10:**
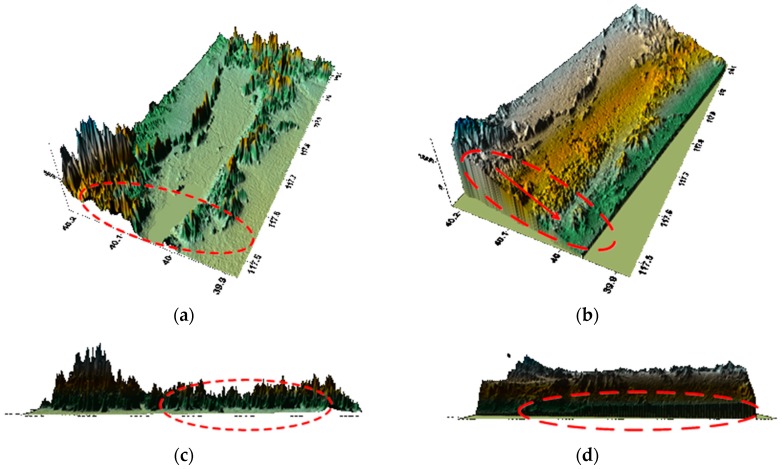
DEM product after orbit fitting: (**a**) SRTM DEM along the latitude; (**b**) GF-3 generated DEM along the latitude; (**c**) SRTM DEM along the longitude; (**d**) GF-3 generated DEM along the longitude.

According to Figure 9, the baseline error varies greatly on account of the poor accuracy of the real-time orbit and Figure 10 shows the DEM generation after orbit fitting by Lagrange polynomial curve fit and the comparison with SRTM DEM.

After orbit fitting, the error changes in latitude approximately linearly as shown in Figure 10b, which facilitates the subsequent baseline error estimation. And there is a slope error in Figure 10d by comparing Figure 10c. Thus the baseline must be calibrated.

#### 3.2.4. Results of Correction

Based on the process flow in Figure 4, we use the fitted orbital information to estimate the elevation error, then the $\Delta B_{∥}$ will be estimated and the orbit will be calibrated. In the end, DEM generation will be carried out and the correction results will be verified by observing the height error.

**Figure 11:**
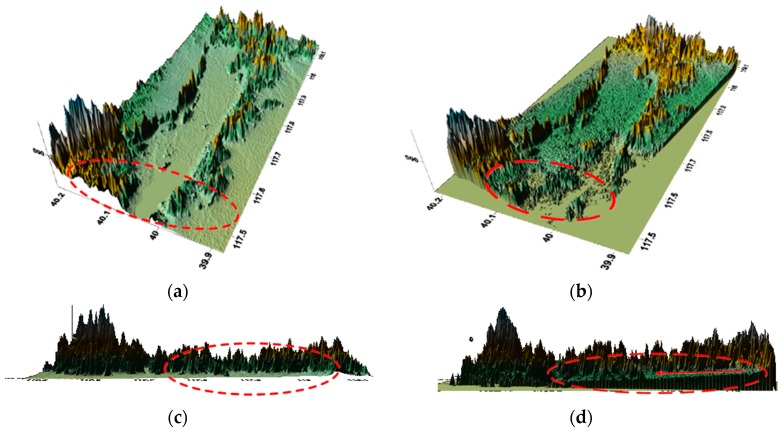
DEM product after correcting $\Delta B_{∥}$: (**a**) SRTM DEM along the latitude; (**b**) GF-3 generated DEM along the latitude; (**c**) SRTM DEM along the longitude; (**d**) GF-3 generated DEM along the longitude.

Figure 11 shows the results when the $\Delta B_{∥}$ is calibrated for the first time and it is observed that the DEM product accuracy has an obvious improvement and the terrain information is fully displayed.

The error along the latitudinal direction has been corrected by comparing Figure 11a,b with Figure 10b. While there is still a slight slope error and Figure 11d displays the difference. Therefore, the baseline must be estimated for the second time to enhance the DEM’s accuracy of convergence. 

**Figure 12:**
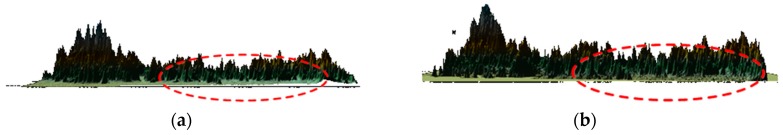
DEM product after correcting $\Delta B_{∥}$ for the second time: (**a**) SRTM DEM; (**b**) GF-3 generated DEM.

Figure 12 shows the result when the error is calibrated for the second time. 

**Figure 13:**
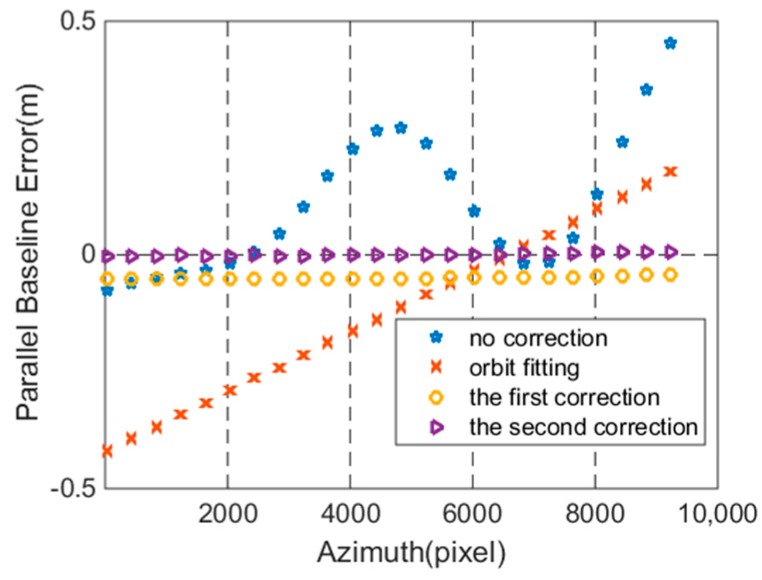
Parallel baseline error comparison.

As can be seen from Figure 12, there is no slope error. Therefore, the modulation error and the slope error have been calibrated after correction. At the same time, we draw the parallel baseline error during the entire process in Figure 13.

From Figure 13, it is evident that the $\Delta B_{∥}$ is around zero after the second correction. The results indicate that the $\Delta B_{∥}$ have been calibrated by SRTM DEM, which benefits the real-time DEM generation.

**Figure 14:**
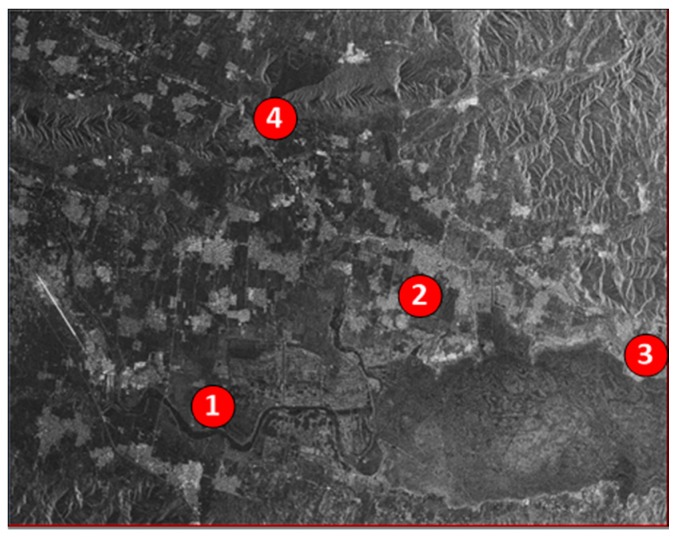
Scattering points in SAR image

Finally, four strong scattering points from the classical land covers, that is, the farmland, the village, the urban area and water body, are selected to demonstrate the high accuracy of DEM generation with the method of baseline estimation. Figure 14 exhibits the positions of the four points in the GF-3 SAR image. 

**Figure 15:**
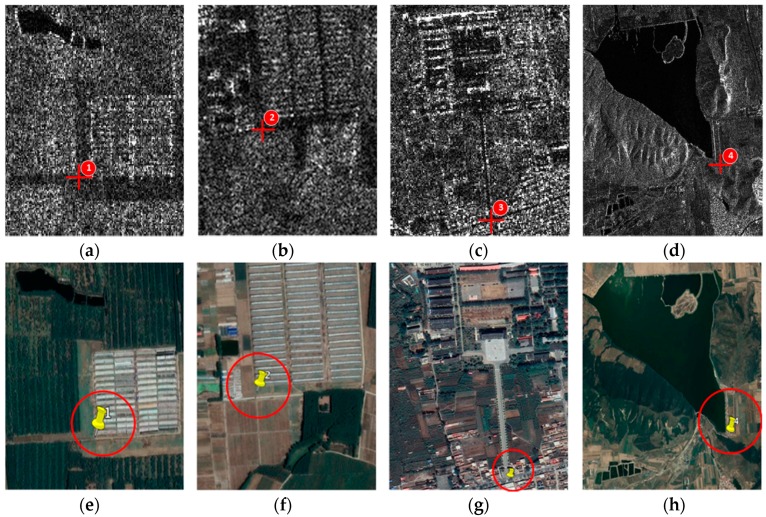
Scattering points: (**a**) the junction of the farmland; (**b**) the crossroad of the village; (**c**) the crossroad of the city; (**d**) the strong scattering point around the water area; (**e**–**h**) four check points on Google Earth.

In order to display the details of the scattering points, the four different aforementioned areas are cropped out after geocoding with the SAR image in Figure 14. From Figure 15a–d, the points are the junction of the farmland, the crossroad of the village, the crossroad of the city and the strong scattering point around the water body. All the points are marked with red cross. Correspondingly, the optical points are pinned up in Google earth map showing in Figure 15e,f. On purpose of presenting the effectiveness of the proposed method, the elevations in Google earth map and the GF-3 generated DEMs of the four points are provided in Table 3.

As shown in Table 3, the elevation errors of the scattering points are within 3 m apart from scattering point 1 in our approach. However, the error can cover tens and hundreds of meters with the satellite orbit state vector method due to the poor accuracy of real-time orbit, which indicates that the approach proposed in the paper is executable for obtaining the terrain information with high accuracy for real-time DEM generation.

## 4. Conclusions

In some special circumstances such as earthquakes, it is necessary to acquire the topographic information quickly. However, the acquisition of scientific orbits requires a certain amount of time and the real-time orbit with poor accuracy will be used for DEM reconstruction, which makes a large baseline error and a low measuring precision. This paper proposes an InSAR baseline estimation method for GF-3 real-time DEM generation based on SRTM DEM. In this experiment, the modulation error has been corrected after the first baseline estimation. However, the slope error still exists so that the error is implemented iteratively to achieve a higher accuracy DEM. After error correction, not only the modulation error and the slope error are calibrated but also the accuracy of the generated DEM is improved significantly. It is worth noting that the error is a constant in traditional methods which is not enough to generate a high-precision DEM. Therefore, the method proposed in this paper obtains the baseline error with a linear least-squares fit, which is equivalent to estimating a linear variation of the error. In the end, four points in Google Earth are used to verify our results and the error is within a few meters, which makes our method more practical for GF-3 real-time DEM generation and the terrain can be reproduced and provide direct data support to help the post-earthquake emergency commanders and staff understand the landscape of the earthquake area.

## Figures and Tables

**Table 1:** Methods comparison.

Methods	Processing			
	Baseline Form	Preprocessing	Baseline Error	Limiting Conditions
Orbit State Vector	Physical baseline and α	No	Constant	Accurate orbits
External Control Points	Physical baseline and α	No	Constant	GCPs
FFT	Physical baseline and α	No	Constant	Flat terrain
Our Method	Parallel baseline	Yes	Linear	No above limits

**Table 2:** The basic Information about the two GF-3 SAR images employed in the experiment.

Parameter	Image #1	Image #2
Region	Hebei	Hebei
Wave length (m)	0.055517	0.055517
Satellite direction Center frequency (GHz) PRF (Pulse Recurrence Frequency) (Hz) Average altitude (m) Look direction Size(Az × Rng) (pixel) Product level Product format	Descending 5.400012 2158.034424 83.756627 Right 9311 × 14,827 1 TIFF	Descending 5.400012 2158.17407 25.202458 Right 9311 × 14,827 1 TIFF

**Table 3:** Results of the checking points.

Scattering Points	Google Earth (m)	Our Method (m)	Satellite Orbit State Vector (m)
1	20	14.1387	74.9312
2	27	25.4709	129.0772
3	81	84.7393	176.2719
4	33	32.6492	144.7998
